# Bacteriophage therapy to combat bacterial infections in poultry

**DOI:** 10.1186/s12985-017-0849-7

**Published:** 2017-09-16

**Authors:** Andrzej Wernicki, Anna Nowaczek, Renata Urban-Chmiel

**Affiliations:** Sub-Department of Veterinary Prevention and Avian Diseases, Institute of Biological Basis of Animal Diseases, Faculty of Veterinary Medicine, University of Life Sciences, Akademicka 12, 20-033 Lublin, Poland

**Keywords:** Bacteriophages, Therapy, Poultry diseases, Antimicrobial resistance

## Abstract

Infections in poultry are an economic and health problem in Europe and worldwide. The most common infections are associated with salmonellosis, colibacillosis, campylobacteriosis, and others. The prevalence of *Campylobacter*-positive poultry flocks in European countries varies from 18% to 90%. In the United States, the prevalence of infected flocks is nearly 90%. A similar percentage of infection has been noted for salmonellosis (about 75–90%) and *E. coli* (90–95%). The occurence of *Clostridium perfringens* is a major problem for the poultry industry, with some estimates suggesting colonization of as many as 95% of chickens, resulting in clinical or subclinical infections. In the US, annual economic losses due to *Salmonella* infections run from $1.188 billion to over $11.588 billion, based on an estimated 1.92 million cases. Similar costs are observed in the case of other types of infections. In 2005 economic losses in the the poultry industry due to mortalities reached 1,000,000 USD.

Infections caused by these pathogens, often through poultry products, are also a serious public health issue.

The progressive increase in the number of multi-drug resistant bacteria and the complete ban on the use of antibiotics in livestock feed in the EU, as well as the partial ban in the US, have led to the growth of research on the use of bacteriophages to combat bacterial infections in humans and animals.

The high success rate and safety of phage therapy in comparison with antibiotics are partly due to their specificity for selected bacteria and the ability to infect only one species, serotype or strain. This mechanism does not cause the destruction of commensal bacterial flora. Phages are currently being used with success in humans and animals in targeted therapies for slow-healing infections. They have also found application in the US in eliminating pathogens from the surface of foods of animal and plant origin. At a time of growing antibiotic resistance in bacteria and the resulting restrictions on the use of antibiotics, bacteriophages can provide an alternative means of eliminating pathogens.

## Background

Bacteriophages are a group of viruses widely distributed in nature whose life cycle is strictly associated with the bacterial cell. They are known as bacterial parasites because they lack the cell structure and enzyme systems necessary for food uptake, protein synthesis or construction of new particles, and as incomplete organisms can only replicate in a live cell.

Bacteriophages were discovered by Twort (1915) as unidentified molecules that inhibit bacterial growth, but in 1917 D’Herelle was the first to isolate and characterize phages, and he also developed the first phage therapy against fowl typhoid induced by *Salmonella* Gallinarum in chickens [1]. Positive results of the use of bacteriophages in fighting bacterial infections have contributed to the development of research on the potential use of viruses that destroy bacteria in treatment of diseases in both human and animals [2, 3].

### Taxonomy of bacteriophages and life cycles

The criterion of taxonomy of bacteriophages applied by the ICTV (International Committee on Taxonomy of Viruses, EC 48, Budapest, Hungary, August 2016) is based mainly on genome type and virion morphology. The ICTV report, based on genomic and proteomic-based methods, was used by the BAVS to classify phages into 873 species, 204 genera and 14 subfamilies in the 2015 taxonomy release [4–6]. The basic classification of the viruses is shown in Table 1. It should be emphasized that the vast majority (about 96%) of known phages belong to the *Myoviridae, Podoviridae and Siphoviridae* [7, 8].

**Table 1:** The basic classification of the viruses based on Virus Taxonomy: 2016 Release EC 48, Budapest, Hungary, August 2016

Order	Family	No of subfamilies	No of genus	No of species
Bunyavirales	Feraviridae		1	1
	Fimoviridae		1	9
	Hantaviridae		1	41
	Jonviridae		1	1
	Nairoviridae		1	12
	Peribunyaviridae		2	52
	Phasmaviridae		1	6
	Phenuiviridae		4	24
	Tospoviridae		1	11
Caudovirales	Myoviridae	6	39 not in a Subfamily	258
	Podoviridae	3	20 not in a Subfam.	121
	Siphoviridae	6	94 not in a Subfam.	522
Herpesvirales	Alloherpesviridae		4	12
	Herpesviridae	3	9	57
	Malacoherpesviridae			2
Ligamenvirales	Lipothrixviridae		3	8
	Rudiviridae		1	3
Mononegavirales	Bornaviridae		1	8
	Filoviridae		3	7
	Mymonaviridae		1	2
	Nyamiviridae		3	4
	Paramyxoviridae		7	49
	Pneumoviridae		2	5
	Rhabdoviridae		18	131
	Sunviridae		1	1
	Unassigned		5	5
Nidovirales	Arteriviridae		5	17
	Coronaviridae	2	6	39
	Mesoniviridae		1	7
	Roniviridae		1	1
Picornavirales	Dicistroviridae		3	15
	Iflaviridae		1	15
	Marnaviridae		1	1
	Picornaviridae		35	80
	Secoviridae		1 Subfam. and 5 not	23
	Unassigned		1	4
Tymovirales	Alphaflexiviridae		7	50
	Betaflexiviridae	2	11	89
	Gammaflexiviridae		1	1
	Tymoviridae		3	39
Unassigned	84 families see full name https://talk.ictvonline.org/taxonomy/	12	361	2488

Their fundamental characteristic is the presence of one type of nucleic acid as a carrier of genetic information and a capsid built from structural proteins. In terms of DNA structure, phages can be divided into three groups: those containing DNA in the form of a double helix, those with a single strand of DNA, and phages containing RNA. Most known bacteriophages have a genome consisting of double-stranded DNA. Two types of bacteriophages are distinguished on the basis of capsid symmetry: isometric (polyhedral) and helical (spiral).

Estimates suggest that bacteriophages are the most abundant life forms on Earth. By 2017 more than 25,000 bacteriophage nucleotide sequences had been deposited in INSDC databases [5, 9]. The common occurrence of bacteriophages is a significant factor facilitating their acquisition and characterization of their suitability for combating bacterial infections. Phages are isolated from all natural environments, including wastewater, human and animal waste, natural water bodies, soil, forest groundcover, food products, and other microorganisms [10–12].

Replication of bacteriophages is similar in many ways to that of eukaryotic viruses. Both involve adsorption, penetration, replication of nucleic acids, formation of virions, and their release from the host cell. Bacteriophages are specifically associated with a particular bacterial strain and exhibit strong bactericidal activity against Gram-positive and Gram-negative bacteria. Some phages display specific affinity for single types of bacteria, while others have a broad range of activity. Their specificity and range of activity is determined by the presence of receptors located on the surface of bacterial cells, among which we can distinguish LPS fragments, fimbriae and other surface proteins [8, 13–15].

We distinguish two types of activity against the bacterial cell: lytic activity, which is characteristic of virulent phages, and lysogenic activity, involving integration of the genetic material of the bacteriophage with the bacterial chromosome and replication as part of the bacterial DNA, resulting in the appearance of a prophage [15].

**Fig. 1:**
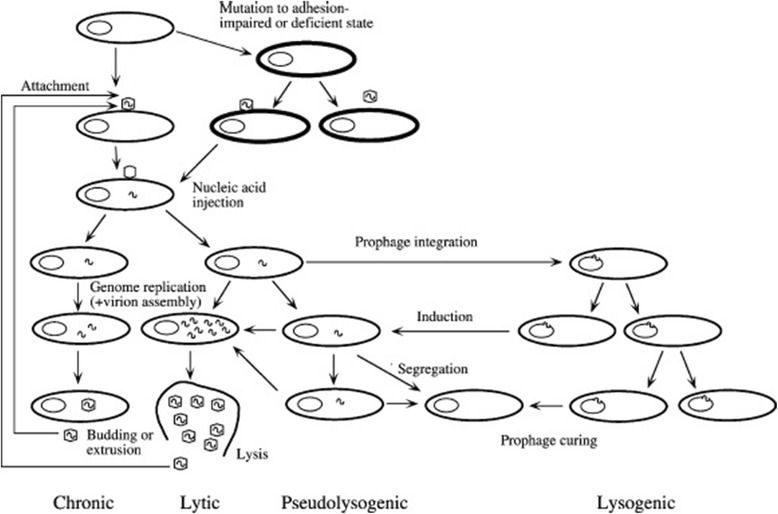
Different types of bacteriophage infection [8]

The lytic cycle of bacteriophages consists of adsorption, which involves adhesion to the bacterial cell, and binding of phage proteins to previously recognized receptors on the bacterial cell surface, such as teichoic and lipoteichoic acid for Gram-positive or LPS for Gram-negative bacteria [14]. The penetration phase involves rupture of the cell wall by the bacteriophage enzymes and penetration of the genetic material into the host cell. Next is the eclipse phase, involving replication of nucleic acid and proteins constituting the structural part of the capsid, while replication of the bacterial DNA is inhibited. This is followed by the formation and maturation of the bacteriophage, lysis of the bacterial cell and the release of daughter phages capable of infecting other cells [8] (Fig. 1). Examples of bacteriophages undergoing the lytic cycle are phages T1 and T4 [16].

The lysogenic cycle involves direct integration of genetic material with the bacterial chromosome, integration with the host genome and formation of the prophage. The replication of the bacteriophage is blocked and its genome enters a state of latency. This state can be interrupted spontaneously or as a result of activation by sunlight, UV radiation, alkylating agents, or certain antibiotics, such as mitomycin C [8, 11] (Fig. 1). Examples of bacteriophages with a lysogenic cycle include λ *Escherichia coli*; Mu, with activity against *E. coli*, *Salmonella*, *Citrobacter* and *Erwinia*; MM1 *S. pneumoniae*; and *φ*11 *S. aureus* [12, 16].

Depending on environmental conditions and the type of bacterial cell, there are several different pathways of bacteriophage infection, including chronic infection, pseudolysogeny and abortive infection (Fig. 1). Not all of these cycles end with the death of the bacterial cell and replication of phage particles. In many cases daughter virions are produced without induction of lysis of the bacterial cells, and thus viral particles are not released outside the cell [8, 17, 18].

### Bacteriophages for control pathogens

The most common bacteria inducing foodborne infections in humans include bacteria of the genera *Salmonella* and *Campylobacter* and *E. coli*. According to the 2015 EFSA report on resistance to antibacterial agents in selected zoonotic bacteria (*Salmonella* and *Campylobacter*), indicator bacteria (*E. coli* and *Enterococcus* spp.), and other bacteria isolated from poultry and from food, a considerable percentage of the isolates posing a threat to humans and animals are resistant to available antibiotics, partially as a result of their widespread use in treatment of disease in humans and animals. The use of bacteriophages to eliminate pathogens seems quite promising, especially as they are present in every ecosystem and number 10^31^, which is more than 10 times the number of characterized bacteria [11, 19, 20].

The effectiveness and safety of phage therapy in comparison to antibiotics is partially due to the specificity of bacteriophages for particular bacteria, manifested as the ability to infect only one species, serotype or strain. This mechanism of action does not cause destruction of the commensal intestinal flora. Self-replication of bacteriophages takes place during treatment, which eliminates the need to apply them repeatedly. Another advantage of phages is that they cannot bind to and replicate in eukaryotic cells, which causes a decrease in their titre, correlated with a marked reduction in the number of pathogenic bacteria inducing a given infection in the organism. An equally important advantage is that phages are not toxic, because most of them are composed mainly of proteins and nucleic acids [21].

Despite its numerous advantages, the use of phage therapy is substantially limited, partly because single bacteriophages cannot be used to combat broad-spectrum infections. In many cases complex identification and characterization of the aetiological agent is necessary. Moreover, not all bacterial viruses meet the criteria for use in treatment, particularly lysogenic phages, which encode genes of bacterial toxins and thereby transform harmless bacteria into pathogenic ones. They can also be involved in transferring drug-resistance genes among bacteria. Another adverse phenomenon in phage therapy is that phages can be cleared by the reticuloendothelial system, reducing their half-life in the organism and limiting the effectiveness of treatment [18, 19, 22].

The increased use of treatment with bacteriophages is determined by their ability to lyse infected bacteria and mutate resistant bacteria, as well as by the high specificity of phages for particular bacteria. A vast number of infections in humans are induced by multi-drug resistant hospital strains of bacteria and by bacteria which have acquired resistance traits in the natural environment. Phage therapy has found application in treating bacterial infections in dermatology, stomatology, otolaryngology, ophthalmology, gynaecology, paediatrics, gastroenterology, urology and pulmonology [23]. The use of bacteriophages in treating infections in humans has had a high success rate (about 85%), particularly in the case of mixed infections induced mainly by *Staphylococcus aureus*, *Klebsiella*, *Escherichia coli*, *Proteus*, *Pseudomonas*, *Enterobacter* and vancomycin-resistant *Enterococci* [24, 25].

### Application of phages in biocontrol and therapeutic design

Phage therapies are also an effective tool in eliminating bacterial infections in various species of animals. Bacteriophages have also proven successful in treating diseases in poultry. One of the objectives of phage therapy in animals is to assess the suitability of bacterial viruses for control of pathogens having an important influence on animal productivity and health. Phages used in treatment have been effective in preventing infections and in treatment of colibacteriosis in poultry [26]. Positive results, with a high success rate in eliminating pathogens, have also been obtained in combating infections induced by various *Salmonella* serotypes in gamefowl, such as Enteritidis and Typhimurium [27–32], as well as campylobacteriosis in poultry, particularly infections induced by *Campylobacter jejuni* and *C. coli* [33]. The effectiveness of phage therapy has also been confirmed in infections of broiler chickens by anaerobic *Clostridium perfringens* during the course of necrotic enteritis [34].

#### Salmonellosis

The therapeutic effectiveness of phages is determined by their high lytic titre, the form and type of application, and the application period. Long-term use of phages in poultry has proved to be moderately effective in reducing the number of *Salmonella* pathogens colonizing the digestive tract [27]. However as shown by Fiorentin et al. [28], single oral application of a cocktail of phages (CNPSA1, CNPSA3 and CNPSA4) at a dosage of 10^11^ PFU decreased the occurrence of *Salmonella* Enteritidis strains by 3.5 log units. The authors confirmed that applying a single dose of a bacteriophage suspension with a high titre was highly effective in reducing the population of pathogenic bacteria in the digestive tract, in contrast with long-term application of a lower titre.

A positive effect of phage therapy was also observed in combating horizontal infections induced by strains of *S.* Gallinarum in flocks of laying hens. Treatment using bacteriophages as a feed additive for chickens having contact with infected individuals led to a mortality rate of only 5%, as compared to 30% in the group that did not receive phage therapy [32].

The effectiveness of phage therapy may also depend on the individual antibacterial properties of a given bacteriophage and on the adaptive mechanisms of the bacteria. A study by Andreatti Filho et al. [30] showed that the use of selected bacteriophages in an orally administered cocktail to prevent colonization by *S.* Enteritidis strains in poultry was only effective for a short time (about 48 h), with no long-term protective effect, which was partly due to acquisition of resistance to the bacteriophage by the bacteria. All treatments resulted in a significant, 6-log reduction in *Salmonella* Enteritidis strains recovered from caecal tonsils at 24 h as compared to untreated controls, but no significant differences were observed at 48 h following treatment.

It seems promising that a wide range of lytic activity against three serovars of *Salmonella* – Enteritidis, Typhimurium, and Hadar – were obtained in 36-day-old broiler chicks, in which a significant reduction in the concentration of bacteria was noted following experimental infection with these serovars, by 2–4 log units [29]. The authors suggest that adjustment of treatment conditions may make it possible to use just one or two bacteriophages rather than many. In another study, Ahmadi et al. [35] demonstrated 100% efficacy in eliminating *S*. Enteritidis strains from the tonsils of 33-day-old *Salmonella*-free quails, 6 h after oral application of 100 ml of a 10^9^ to 10^10^ PFU ml^−1^ bacteriophage suspension. It should be noted that all birds received the bacteriophage suspension for 3 days, and the therapeutic effect was noticeable within 6 h after experimental infection. The authors also confirmed that this treatment has a prophylactic effect in quails receiving 100 μl of 10^6^ PFU ml^−1^ bacteriophages via oral gavage for 3 days, once every 24 h, before oral challenge with 100 μl of 1.2 × 10^9^ CFU ml^−1^ *S*. Enteritidis. Significant prevention of colonization by *S*. Enteritidis strains was observed over a period of 7 days at a rate of 20% in comparison to the control (100% colonization).

Other studies suggest that bacteriophages may be used in combined treatment with other preparations, as indicated by a significant (about 80%) synergistic antibacterial effect of a commercial oral probiotic preparation applied together with a bacteriophage ‘cocktail’ of phages S2a, S9, and S11 (5.4 × 10^6^ PFU/0.5 ml/bird) at 4, 5, and 6 days of age and at 8, 9, and 10 days of age to combat *S.* Typhimurium infections in broilers. The authors showed that chickens treated with a probiotic and bacteriophages showed 10 times fewer bacteria in the ileum, caecum, liver and spleen than did untreated challenged chickens. [31].

In another study, simultaneous application of three phages (MOI 103) at 10^8^ PFU/ml/dose at 6 days of age (two daily doses) by aerosol spray and probiotics administered at 1 day of age by coarse spray, followed by oral inoculation with 2.95 × 10^5^ CFU/ml in seven-day-old chickens, reduced *Salmonella* incidence and Salmonella intestinal colonization, leading to complete elimination of deaths in broiler chickens caused by infection with *Salmonella* Enteritidis [36]. Similar results were obtained in inhibiting horizontal infection with *Salmonella* following application of a bacteriophage suspension in the amount of 10^5^ and 10 PFU/g as a feed additive for chickens challenged with 5 × 10^7^ CFU of bacteria. Different groups of birds were treated with different titres of bacteriophage contained in the feed additive for 21 days after *Salmonella* Enteritidis challenge. These preventive measures significantly inhibited the replication of pathogens in the digestive tract of the chickens; however, this effect was observed mainly in chickens treated with bacteriophages at concentrations of 10^9^ PFU/mL, which were compared only with the positive control groups [37]. Authors also suggest the occurrence the horizontal transmission of *Salmonella* Enteritidis strains, which was confirmed by a substantial reduction in the number of chickens treated with bacteriophages at concentrations of 10^7^ and 10^9^ PFU/g 1 week after treatment in comparison with untreated chickens. However, there was no significant reduction in *Salmonella* counts after two and 3 weeks of treatment as compared with the positive control group. And in many cases, the efficacy of phage therapy should be maximized by using a high titre of bacteriophages in order to reduce colonization by *Salmonella* via passive transmission.

#### Colibacillosis

Phage therapy has also proven to be an effective therapeutic tool in fighting pathogenic strains of *Escherichia coli*, particularly in preventing the development of colibacillosis, which initially develops in the respiratory tract and air sacs and then takes the form of sepsis, causing considerable mortality in poultry.

Phage suspensions applied directly to the air sac in 3-day-old birds in a range of titres from 10^6^ to 10^3^ PFU to treat *E. coli* infections substantially reduced mortality rates to 5% and 25%, respectively. Similar results were obtained after inoculation of a bacteriophage suspension in the drinking water of birds at 1 week of age (10^3^ or 10^4^ PFU of bacteriophages per mL) followed by air sac challenge with 10^3^ CFU of *E. coli* phages. Mortality was decreased to 25% and 5%, respectively. No mortality was observed in chickens treated with 10^8^ PFU of an *E. coli* bacteriophage mixture [38]. Bacteriophages have also been shown to be highly effective in treating sepsis and meningitis in newly hatched and 3-week-old chicks infected intramuscularly and intracranially with a strain of *E. coli*. Mortality in the untreated chicks was 100%, whereas intramuscular administration of phage R at titres of 10^4^ and 10^6^ PFU completely eliminated deaths in chickens in the group of treated birds. Another positive effect of the treatment was the absence of visible clinical symptoms. In the chickens intracranially infected with *E. coli*, application of a higher dose of the phage, at a titre of 10^8^ PFU, fully protected the birds against the development of infection. Intramuscular application (in different muscles) of phage R at a titre of 10^6^ PFU resulted in a lack of morbidity or mortality in all chickens. Administration of lower doses from 10^4^ PFU of the phage after challenge with *E. coli* also provided significant protection, indicating that the phage had multiplied in vivo. However, the application of phages in lower doses, e.g. 10^2^ PFU, produced no statistically significant protection against *E. coli* infection.

The authors also demonstrated that the bacteriophages administered to the birds intramusculary had the ability to penetrate the blood-brain barrier, and confirmed that the bacteriophages had a prophylactic effect in addition to the therapeutic effect. In 3-week-old birds effective protection against morbidity and mortality following intracranial inoculation with *E. coli* was obtained only after administration of 10^8^ PFU of the phage. Only in younger birds was statistically significant protection obtained after administration of 10^6^ PFU of the phage. Application of a suspension 1–2 days prior to experimental infection with *E. coli* in chicks reduced the mortality rate by 70%, as well as the intensity of the course of infection [26]. The use of bacteriophages at titres of 10^4^–10^2^ PFU in the form of an aerosol in chicks with symptoms of colibacillosis significantly reduced the mortality of the chicks and prevented infections in other birds. Aerosol administration of bacteriophage SPR02 at a titre of 10^8^ PFU/mL combined with challenge with 10^4^ CFU/mL of *E. coli* completely protected the birds against infection. When these phages at 10^4^ PFU/mL were mixed with 10^4^ CFU/mL of *E. coli*, mortality was significantly reduced to 35%^.^

The authors suggest also that similar effects preventing early development of colibacillosis in chicks are obtained by applying a bacteriophage suspension *in ovo* [39]. The authors also demonstrated that the effect of this kind of bacteriophage treatment is comparable to enrofloxacin treatment, and suggest that a combination of enrofloxacin and bacteriophage treatments could be efficacious and beneficial in controlling colibacillosis.

Apart from bacteriolytic activity, the effectiveness of bacteriophages is also determined by the site and route of administration of the preparation. According to Huff et al. [40] bacteriophages should be applied directly to the site of infection, which was confirmed during treatment of *E. coli* infections in the air sacs of chickens. Application of bacteriophages per os with drinking water proved ineffective in treating the infection and reducing clinical symptoms. When a suspension was injected directly into the air sac, an effective protective effect was obtained, manifested as the absence of clinical symptoms. This treatment significantly reduced mortality from 50 to 20% when given immediately after challenge, but had little efficacy when administered 24 or 48 h after challenge. IM injection of bacteriophages significantly reduced mortality from 53 to 17%, 46 to 10%, and 44 to 20% when given immediately, 24 h, or 48 h after challenge, respectively.

A similar effect eliminating disease symptoms in *E. coli* respiratory infections in poultry was obtained in broiler chickens aged 10 days to 2 weeks following repeated application of a two-phage (SPRO2 and DAF6) suspension in aerosol spray form after challenge with *E. coli* by injection of 10^4^ CFU into the thoracic air sac. The authors observed the best overall protection after aerosol treatment with phage titres of 2.6 × 10^8^ and 2.35 × 10^9^ PFU/mL for SPR02 and DAF6, respectively. The study found a significant decrease in mortality ranging from 20% and 27% in comparison to chickens untreated with bacteriophages, but mortality was still high [40]. In the septic form of colibacillosis intramuscular application proved more effective than aerosol application, particularly in the initial phase of sepsis. In another study on broiler chicks, Huff et al. [41] demonstrated a prophylactic effect of application of a bacteriophage suspension in the form of a single intramuscular injection of two different bacteriophages (10^9^ PFU/mL) in combination with enrofloxacin administered in drinking water immediately after *E. coli* challenge. Mortality decreased significantly to 15% in comparison with untreated birds challenged with *E. coli* (68%). The authors also demonstrated a significant synergistic protective effect in chickens that received both the bacteriophage and enrofloxacin. Because colibacillosis in poultry develops in the respiratory system, some studies suggest that bacteriophages should be applied in aerosol form as a preventive measure 1–3 days before anticipated infection, e.g. transport or transfer to a new environment [42]. The incidence of disease in 7-day old chicks treated with a phage aerosol in the first few days following experimental infection fell to under 10%, while the mortality rate in the chicks that did not receive the aerosol was 60%. A study by Oliveira et al. [43] confirmed that colibacillosis-induced high morbidity and mortality in poultry may be significantly reduced by aerosol spraying of housing systems with bacteriophage cocktails and oral administration of bacteriophages. In that study 1 ml of the phage suspension at a high titre of 1.0 × 10^9^ PFU/ml and a lower titre of 5.0 × 10^7^ PFU/ml of phages phi F78E, F258E and F61E was dispensed into the birds’ mouth with a syringe and by spraying directly into the beak, through a spray nozzle set to release 1 ml per fine drop scatter. Immediately after phage administration the chickens were challenged with a pathogenic *E. coli* suspension. The results also demonstrated a protective effect of the bacteriophages against new colonization by *E.coli* strains in the days following the challenge. The study confirmed the therapeutic effectiveness of one of the phages, phi F78E, administered orally and by spray at 1 × 10^9^ PFU/mL, resulting, on average, in a 25% decrease in mortality and a 41.7% reduction in morbidity in the chickens.

The direct or aerosol administration of bacteriophages in poultry and evaluation of their therapeutic effect has been a subject of study at many research centres. A study by El-Gohary et al. [44] demonstrated that bacteriophage treatment of litter by spraying 200 mL of a bacteriophage preparation at a titre of 8 × 10^8^ PFU/mL on the surface of 3.9 m^2^ pens significantly reduced the mortality of male broiler chickens (about 2–3 weeks old) with colibacillosis resulting from exposure to *E. coli* in the litter, even when the birds were exposed to cold stress, and furthermore reduced shedding of the pathogen among flocks.

#### Campylobacteriosis

The potential uses of phage therapy against *Campylobacter* bacteria may offer an alternative means of eliminating bacteria in the digestive tract of birds. This pertains in particular to infections induced by *Campylobacter jejuni* and *C. coli*, which constitute 80% of the bacteria colonizing the digestive tract in poultry. One of the first attempts to use bacterial viruses against *Campylobacter* bacteria was a study by Wagenaar et al. [33], in which colonization by *C. jejuni* was inhibited in 10-day-old chicks and adult birds, first by 2 and then by 1 log unit in broiler caeca. The Ross broiler chickens received phages by oral gavage from day 7 to 16 at different titres varying from 4 × 10^9^ to 2 × 10^10^ PFU and were orally challenged with 1 × 10^5^ CFU of *C. jejuni* on day 10.

The authors confirmed that phage treatment prior to bacterial challenge does not prevent but may delay bacterial colonization. However, chicks receiving phages after *C. jejuni* colonization showed an immediate 3-log reduction in CFU counts. It should be emphasized that the bacteria were not completely eliminated in this study, which is the main problem in the use of phage therapy for elimination of *Campylobacter* strains in poultry [33].

In another study, conducted at the University of Nottingham in the UK on 25-day-old chickens, following application by oral gavage of bacteriophages CP34 or CP8 isolated from the environment against *C. jejuni* strains HPC5 and GIIC8 obtained from birds and humans, a substantial but short-lived reduction in the number of bacteria was obtained in the intestines of infected birds, ranging from 0.5 to 5 log units. A considerable reduction in the total number of *Campylobacter* bacteria in the upper and lower digestive tract and in the caecum was obtained when bacteriophages were applied at a concentration of 10^7–9^ PFU [45]. A study evaluating the effect of bacteriophages on the number of *Campylobacter jejuni* in the caecum in broiler chickens also confirmed a significant (*P* < 0.001) reduction in the total number of bacteria, to a value of 10^5.1^ CFU/g, in comparison with chickens that were not treated with bacteriophages (mean density of bacteria 10^7^ CFU/g) [46]. A similar reduction in the number of *Campylobacter jejuni* and *Campylobacter coli* bacteria in infected birds was obtained following application of a suspension of bacteriophage CP220 at titres of 10^7^ and 10^9^ PFU/ml for 5 days. A reduction in *C. jejuni* bacteria was observed just 48 h after injection of the phage, while in the case of *C. coli* a significant decrease in the number of infected birds was obtained following administration of a bacteriophage suspension with a density of 10^9^ PFU. It should be emphasized that the percentage of birds resistant to a second *Campylobacter* infection was very low, at about 2% [47].

The use of a suspension of bacteriophages specific for *Campylobacter jejuni* and *C. coli* bacteria in the water or feed of broiler chickens caused a significant decrease of nearly 2 log_10_ CFU/g in colonization by both species of bacteria. Moreover, in contrast with earlier research, the bactericidal effect of the phages was maintained for over 7 days, enabling application of the suspension at each stage of the production cycle [48]. Preventive treatment delayed but did not prevent colonization. Levels of *C. jejuni* were initially 2 log units lower than in controls, and then stabilized at 1 log unit lower than in the controls.

On the other hand, the use of bacteriophages to prevent colonization by *Campylobacter* spp. bacteria in newly hatched broiler chicks was only partially successful. Application by oral gavage of a phage suspension with 0.4 to 2 × 10^10^ PFU/mL of phage 71 in 10-day-old broiler chickens initially reduced the total number of bacteria, but colonization by pathogens re-occurred within 24 h [33]. The studies cited also showed that resistance of *Campylobacter* spp. to particular phages was about 4%. For this reason the authors suggest creating a combination of several bacteriophages specific for *Campylobacter*, which in vitro research has shown to improve the effectiveness of phage therapy [49].

#### Clostridiosis and listeriosis

Phage therapy was shown to be effective in the case of infection of broiler chickens with anaerobic *Clostridium perfringens* inducing necrotic enteritis [34]. Bacterial toxins produced by this bacterium are responsible for generalization of the disease process, cause a decrease in feed intake, and inhibit growth. Oral administration to chickens of various ages of a suspension of a cocktail (INT-401) of 5 different *C. perfringens* phages (CPAS-7, CPAS-12, CPAS-15, CPAS-16, and CPLV-42) at titres of 10^5^ PFU/mL, with feed or water or by oral gavage and spray application, led to a significant decrease (*P* ≤ 0.05) in mortality during the 0-to-42-day of experiment in comparison with the group of untreated birds. These measures also improved weight gain in the chicks. It should also be emphasized that the treatment proved more successful in reducing mortality than a formalin-inactivated vaccine containing *C. perfringens* alpha toxin. However, the study cited confirmed the high efficacy of the bacteriophages in controlling necrotic enteritis in poultry.

Besides whole phages, phage enzymes (endolysins and mureolytic enzymes), including murein hydrolase, merit particular attention as an additive element in combating infections induced by *C. perfringens*. These enzymes, binding directly to the peptidoglycans of the cell walls of Gram-positive bacteria, cause rapid lysis of these bacteria, including cells infected with bacteriophages, which accelerates their destruction. Simultaneous use of bacteriophage preparations and endolysins against Gram-positive bacteria such as *Clostridium* spp. and *Listeria monocytogenes* seems to have a highly beneficial effect. This has been confirmed in the case of use of a bacteriophage (ϕ3626) against *C. perfringens*, whose spectrum of lysogenic activity was at a level of 22%, while the lytic effect was 8%. In the case of combined application of bacteriophages with murein hydrolase, a lytic effect was observed against all (*n* = 51) tested strains of *C. perfringens* [50, 51]. In view of the fact that bacteriophages eliminate *C. perfringens* bacteria mainly by lysogeny, supplementation of phage preparations with endolysins would seem to be necessary for continuation of successful treatment.

The bactericidal effectiveness of phages has also been confirmed in fighting infections induced by *Listeria monocytogenes*, which like *Campylobacter* spp. or *Salmonella* is included among zoonotic pathogens inducing food poisoning in humans, with a high mortality rate of 30%. Application of bacteriophages on the surface of poultry products ready for consumption reduced the number of bacteria by 2.5 log units on a product stored at 30 °C after just 5 h. Later testing for *Listeria monocytogenes* in food samples kept in cold storage also yielded positive results, as the pathogen was not detected for a period of 21 days or use of a bacteriophage mixture on poultry carcasses could completely eliminated *L. monocytogenes* [52, 53]. Due to the risk posed by the occurrence of poultry infections induced by *L. monocytogenes*, as well as their increasing drug-resistance and efforts to limit the use of antibiotics, international and American health organizations are attempting to replace antibiotics with other preparations. This resulted in FDA approval on 18 August 2006 of 102-LMP™, a suspension of bacteriophages specific for *L. monocytogenes*, as an antibacterial agent against *L. monocytogenes*. This product has been estimated to successfully kill over 170 strains of *Listeria* spp. [54].

### The main obstacles to the use of phage therapy in poultry

﻿The full summary about using of bacteiophages in poultry’s experimental activities are included in Table 2. ﻿Despite the significant positive aspects of phage therapy, there are also some limitations in the widespread use of bacteriophages to eliminate pathogens. One of the main obstacles to elimination of bacteria from poultry is that significant numbers of phages are needed to adsorb individual host cells [50]. Some authors [38] have shown that the application of phages in lower doses, e.g. 10^2^ PFU, provided no statistically significant protection against *E. coli* infection. Moreover, preventive treatment in phage therapy did not prevent colonization [48].

**Table 2:** Summary of studies on phage therapy in bacterial infections in poultry

Animals	Objective	Challenge	Phage application	Observations	Reference
One-day-old chickens	Reduce contamination of poultry products by food-borne pathogens; reduce morbidity, disease severity and mortality	Oral inoculation with 100 μl fresh *S*. Enteritidis PT4 culture at 10^8^ CFU/bird	Single oral application of phage cocktail (CNPSA1, CNPSA3 and CNPSA4) at 10^11^ PFU	1. A single dose of a bacteriophage suspension with a high titre was highly effective in reducing the population of pathogenic bacteria in the digestive tract. 2. Reduction of 3.5 orders of magnitude in colony-forming units of *S*. Enteritidis PT4 per gram of caecal content	Fiorentin et al. 2005
6-week-old chickens	Reduce morbidity, disease severity and mortality	Oral challenge with *S.* Gallinarum at 5 × 10^8^ CFU/mL	Bacteriophage CJø01 as feed additive at 10^6^ PFU/kg	1. Treatment using bacteriophages as a feed additive for chickens having contact with infected individuals led to a mortality rate of only 5%, as compared to 30% in the group that did not receive phage therapy	Lim et al. 2011
One-day-old chickens	Reduce morbidity, disease severity and mortality	Challenge with *S. Enteritidis* by oral gavage (0.25 mL) at 9 × 10^3^ CFU/chick	Cocktails of 4 different bacteriophages obtained from commercial broiler houses (CB4∅) and 45 bacteriophages from a municipal wastewater treatment plant (WT45∅), administered by oral application at 10^8^ PFU/chick	1. Bacteriophages as cocktail in oral administration to prevent colonization by *S.* Enteritidis strains in poultry were only effective for a short time (24–48 h) with no long-term protective effect	Andreatti Filho et al. 2007
36-day-old chickens	Reduce morbidity, disease severity and mortality	Challenge with 1 ml of an 8.0-log 10 CFU/ml^−1^ suspension of *S.* Enteritidis	Bacteriophage 151 against *S.* Enteritidis, bacteriophage 25 against *S*. Hadar, bacteriophage 10 against *S*. Typhimurium. Bacteriophage suspensions administered by oral gavage at 10^9^ PFU/ml and 10^11^ PFU/ml)	1. Significant reduction in the concentration of two of three serovars (*S.* Enteritidis, Typhimurium) by 2–4 log units after administration of a bacteriophage suspension with a density of 10^11^ PFU	Atterbury et al. 2007
33-day-old quails	Efficiency of bacteriophage administration in prophylactic and therapeutic contexts	Oral challenge with 100 ml of *S*. Enteritidis at 1.2 × 10^9^ CFU/mL	Single Salmonella-lysing phage (PSE) at 10^9^ PFU/ml in 100 μl aliquot by oral gavage for 2 days	1. 100% efficacy in eliminating *S*. Enteritidis strains from the tonsils, 6 h after application of bacteriophage suspension 2. PSE phage was more effective when administered prophylactically prior to *S.* Enteritidis infection than as a treatment for established *S.* Enteritidis infections.	Ahmadi et al. 2016
One-day-old chickens	Reduce morbidity, disease severity and mortality	Oral challenge with 0.5 ml of a suspension of S.Typhimurium at 2.4 × 10^5^ CFU/mL or 7.9 × 10^5^ CFU/mL	Bacteriophage cocktail (S2a, S9, S11), at an oral dosage of 10^6^ PFU/bird on days 4–6 and 8–10 of age	1. 10-fold reduction in bacteria in chicken ileum, caeca, liver and spleen 2. Synergistic antibacterial effect of oral commercial probiotic preparation applied together with a bacteriophage cocktail	Toro et al. 2005
One-day-old chickens	Reduce morbidity, disease severity and mortality	Oral challenge with 2.95 × 10^5^ CFU/mL *S.* Enteritidis	Cocktail of 3 phages by aerosol spray at 10^8^ PFU/ml/dose for each phage at 6 days of age (two daily doses) and probiotics administered at 1 day of age by coarse spray	1. Effective method for reducing *S*. Enteritidis colonization in intestinal chickens, leading to complete elimination of deaths in broiler chickens caused by infection with *S.* Enteritidis	Borie et al. 2009
One-day-old chickens	Reduce morbidity, disease severity and mortality	Oral challenge with *S.* Enteritidis at 5 × 10^8^ CFU/mL	Bacteriophage CJ07 as a feed additive at three concentrations (10^5^, 10^7^ and 10^9^ PFU/g) for 21 days after challenge with *S.* Enteritidis	1. Highest doses of bacteriophage significantly inhibited the replication of pathogens in the digestive tract of the chickens. 2. The efficacy of phage therapy should be maximized by the use of a high titre of bacteriophage to reduce *Salmonella* colonization by passive inundation.	Lim et al. 2012
3-day-old birds 7-day-old birds	Reduce morbidity, disease severity and mortality	*E. coli* by injection of 10^4^ CFU/mL into the thoracic air sac at 7, 8, or 10 days of age	Bacteriophages SPR02 applied directly to air sac in a range of titres from 10^8^ to 10^3^ PFU Bacteriophage suspension applied to drinking water (10^3^ or 10^4^ PFU/ml)	1. Reduced mortality rates to 5% and 25% and 100% depending on the titre of bacteriophage suspensions 2. Reduced mortality rates to 5 and 25% depending on the titre of bacteriophage suspensions	Huff et al. 2002
3-week-old chickens	Reduce morbidity, disease severity and mortality	*E. coli* O1:K1 or O2:K1 10^6^ CFU/mL by intramuscular inoculation	Bacteriophage R intramuscularly (10^8^ PFU and 10^6^ PFU)	1. Good protection against morbidity and mortality following intracranial/intramuscular inoculation with *E. coli* 2. The phage was able to multiply in the blood.	Barrow et al. 1998
3-day-old birds	Reduce morbidity, disease severity and mortality	*E. coli* 6 × 10^4^ CFU/mL by injection into the thoracic air sac	Bacteriophage suspension via aerosol spray (10^8^ PFU/ mL, 10^4^ PFU/mL)	1. Significantly reduced mortality (100%) and morbidity (35%) depending on the titre of bacteriophage	Huff et al. 2009
10-day-old birds	Reduce morbidity, disease severity and mortality	Injection of 10^4^ CFU/mL of *E. coli* into the thoracic air sac	Bacteriophage cocktail (10^8^ PFU/mL DAF6, 10^9^ PFU/mL SPR02) in aerosol spray Bacteriophage cocktail (10^9^ PFU/mL DAF6, 10^8^ PFU/mL SPR02) - intramuscular application	1. Decrease in mortality ranging from 20% to 27% in comparison with untreated chickens immediately after challenge 2. Significantly reduced mortality independent of time when bacteriophage cocktail was used	Huff et al. 2003
7-day-old birds	Reduce morbidity, disease severity and mortality	Injection of 10^4^ CFU/mL *of E. coli* into the thoracic air sac.	2 different bacteriophages DAF6 and SPR02 (10^9^ PFU/ml) in intramuscular dosage administered immediately after *E. coli* challenge with enrofloxacin administered with drinking water	1. Significantly decreased mortality to 15% in comparison with untreated birds challenged with *E. coli* (68%) 2. Synergistic additive effect of protection in chickens after use of bacteriophage and enrofloxacin together	Huff et al. 2004
10-week-old chickens	Reduce morbidity, isease severity and mortality	0.2 ml of a 3 h *E.coli* (APEC H839E) culture at 5.0 × 10^8^ CFU/mL by injection into the left air sac	Bacteriophage cocktail (phi F78E, F258E and F61E) at two different titres: 10^7^ and 10^9^ PFU/ml) applied in aerosol spray and drinking water in a single application	1. High titre of bacteriophage decreased mortality and morbidity by 25% and 43%, respectively. 2. Effect in prophylactic context depended on titre. 3. Synergistic protective effect after aerosol spraying of housing systems together with administration of bacteriophages per os	Oliveira et al. 2010
Three-week-old chickens	Reduce morbidity, disease severity and mortality	0.5 mL *E. coli* culture containing 4.5 × 10^8^ CFU/mL or 5.5 × 10^8^ CFU/mL/bird	Bacteriophage preparation applied to litter at titre 10^8^ PFU/ml - 200 ml sprayed on surface of 3.9 m^2^ pens after *E. coli* challenge	1. Mortality was significantly reduced by spraying bacteriophage on the litter. 2. Reduction in shedding of *E. coli* among poultry flocks	El-Gohary et al. 2014
10-day-old chickens, 32-day old chickens	Reduce contamination of poultry products by food-borne pathogens and efficiency of bacteriophage administration in prophylactic and therapeutic contexts	Oral dose of 1 × 10^5^ CFU/g *C. jejuni*	Bacteriophage 71 in a range of titres (from 10^9^ to 10^10^ PFU) applied in oral gavage for 10 days; on day 10 oral *C. jejuni* challenge of 10^5^ CFU (prophylactic context) Oral dose of 10^5^ CFU *C. jejuni* on day 10, followed by inoculation with phage strain 71 (from 10^9^ to 10^10^ PFU) for 6 days starting 5 days after *Campylobacter* administration (therapeutic context) First oral dose of 10^5^ CFU *C. jejuni* followed by inoculation with phage strain 71 (from 10^10^ to 10^11^PFU) and phage 69 (from 10^9^ to 10^10^PFU) for 4 days	1. Inhibited *C. jejuni* in 10-day-old chicks and adult birds, first by 2 and then by 1 log unit in broiler caeca. 2. Phage treatment prior to bacterial challenge does not prevent but could delay bacterial colonization. 3. Bacteriophage cocktail used in adult birds resulted in a significant decrease in *Campylobacter* colonization.	Wagenaar et al. 2005
25-day-old chickens	Reduce contamination of poultry products by food-borne pathogens	*C. jejuni* HPC5 log_10_ 2.7 to 7.8 CFU/g by oral gavage (n = four birds per dosage)	Bacteriophage cocktail (CP34 or CP8 from 10^7^ to 10^9^ PFU), application by oral gavage	1. Short-lived reduction in the number of bacteria in the intestines of infected birds, ranging from 0.5 to 5 log units	Loc-Carrillo et al. 2005
	Reduce contamination of poultry products by food-borne pathogens	Challenge with *C. jejuni* HPC5 or *C. coli* OR12 8 log_10_ CFU/mL by oral gavage	Bacteriophage CP220 at titres of 10^7^ and 10^9^PFU/ml, application for 5 days	1. Reduction in *C. jejuni* bacteria after administration of a bacteriophage suspension with a density of 10^9^ PFU 2. Significant decrease in the number of birds infected *C. coli* following administration of a bacteriophage suspension with a density of 10^9^ PFU	El-Shibiny et al. 2009
31-day-old chickens	Reduce contamination of poultry products by food-borne pathogens	*C. jejuni* 2140 CD1 at titre of 2.2 and 1.1, and 5.8 × 10^6^ CFU/g by oral gavage and in feed	Bacteriophage cocktail (phiCcoIBB35, phiCcoIBB37, phiCcoIBB12) at 10^7^ PFU in feed gavage and 10^6^ PFU oral dosage	1. Reduction in titre of both *C. coli* and *C. jejuni* in faeces by approximately 2 log units after oral gavage and in feed administration. 2. 30-fold reduction in the incidence of campylobacteriosis associated with consumption of chicken	Carvalho et al. 2010
One-day-old chickens	Reduce morbidity, disease severity and mortality	*C. perfringens* CP-6 strain 10^8^ CFU/ml at 1.0 ml/bird by oral gavage	Bacteriophage cocktail (CPAS-7, CPAS-12, CPAS-15, CPAS-16, and CPLV-42 at titres of 10^5^ PFU/ml) with feed or water or oral gavage and spray application	1. Significantly reduced mortality of *C. perfringens*-challenged birds by 92%. 2. Weight gain and feed conversion ratios were significantly better in *C. perfringens*-challenged chickens treated with the bacteriophage cocktail.	Miller et al. 2010

In some cases a protective effect was obtained only in younger birds after high (10^6^ PFU) doses of phage administration [26]. In many cases, the efficacy of phage therapy should be maximized by the use of a high titre of bacteriophages to reduce *Salmonella* colonization by passive inundation. An additional obstacle in the use of phage therapy is that colonization of chicken caeca by *S. enterica* serotypes Enteritidis and Typhimurium is inhibited for only 24 to 48 h after phage treatment. For this reason it seems necessary to determine the optimal timing and delivery of bacteriophages in a real-life poultry industry setting [37]. An important problem in phage therapy is that only strongly lytic phages are suitable. An area of safety concern is the potential release of toxic proteins from the lysing bacteria. In some cases, lysing bacteria inside a patient are known to release endotoxins that cause fever, and sometimes toxic shock [55].

Furthermore, the use of a defined phage or phage mixtures with largely uncharacterized genomes seems to be dangerous. Only full characterization and screening of phages can eliminate those that encode toxic proteins or proteins that allow temperate (integrative) phage behaviour. An important disadvantage in terms of safety is immune responses induced by the phage. All phages contain foreign proteins which could induce an immune response potentially reducing the effectiveness of the therapy, or even cause death as a consequence of anaphylactic shock [56, 57].

To increase the safety of bacteriophages in the elimination of pathogens, the following can be recommended: the use of only strong lytic bacteriophages, not lysogenic phages, switching to non-lysing tailocins if toxic proteins released from the bacteria become a problem; the use of rapid DNA sequencing to characterize phages used in therapy; and prescreening of patients for hyper-immune reactions to the specific phage sample prior to injection, especially in whole flocks.

## Conclusion

The increasingly observed acquisition of antibiotic resistance by bacteria necessitates new strategies for combating drug-resistant bacteria. The results of research on bacteriophages, indicating that they can be an alternative means of eliminating pathogens posing a threat to humans and animals, justify its continuation, particularly in view of increasing drug-resistance in bacteria and restrictions on the use of antibiotics. The development of adequate phage preparations may in the future prove to be one of the most effective methods for fighting bacteria that are pathogenic for humans and animals, and will also make it possible to obtain products that are safe and free of antibiotics.

## Abbreviations

CFU: Colony-forming unit
G: Gram
Log: Logarithm
Ml: Milliliter
N: Number
PFU: Plaque-forming unit
